# “Perspectives on financing population-based health care towards Universal Health Coverage among employed individuals in Ghanzi district, Botswana: A qualitative study”

**DOI:** 10.1186/s12913-016-1657-2

**Published:** 2016-08-19

**Authors:** Barnabas Africanus Mbogo, Deborah McGill

**Affiliations:** 1 Ghanzi District Health Management Team, P.O. Box 7, Ghanzi, Botswana; 2 Department of Public Health and Policy, University of Liverpool, Liverpool, L69 3GL UK

**Keywords:** Health coverage schemes, Universal Health Coverage, Health financing, Community perceptions, Botswana, Qualitative study

## Abstract

**Background:**

Globally, about 150 million people experience catastrophic healthcare expenditure services annually. Among low and middle income countries, out-of-pocket expenditure pushes about 100 million people into poverty annually. In Botswana, 83 % of the general population and 58 % of employed individuals do not have medical aid coverage. Moreover, inequity allocation of financial resources between health services suggests marginalization of population-based health care services (i.e. diseases prevention and health promotion). The purpose of the study is to explore perspectives on employed individuals regarding financing population based health care interventions towards Universal Health Coverage (UHC) in order to make recommendations to the Ministry of Health on health financing options to cover population-based health services.

**Methods:**

A qualitative design grounded in interpretivist epistemology through social constructivism lens was critical for exploring perspectives of employed individuals. Through purposive and snowballing sampling techniques, a total of 15 respondents including 8 males and 7 females were recruited and interviewed using a semi-structured format. Their age ranged from 23 to 59 years with a median of 36 years. Data was analyzed using Thematic Content Analysis technique.

**Results:**

Use of social constructivism lens enabled to classify emerging themes into population coverage, health services coverage and financial protection issues. Despite broad understanding of health coverage schemes among participants, knowledge appears insignificant in increasing enrolment. Participants indicated limited understanding of UHC concepts, however showed willingness to embrace UHC upon brief description. Main thematic issues raised include: exclusion of population-based health services from coverage scheme; disparity in financial protection and health services coverage among enrollees; inability to sustain contracted employees; and systematic exclusion of unemployed individuals and informal sector employees.

**Conclusion:**

Increasing enrolment in health coverage schemes requires targeted campaign for information dissemination through use of myriads mass media including: social networks, TV, Radio and others. Moreover, re-designing health insurance schemes is critical in order to include population-based interventions; expand uptake of unemployed and informal sector employees; flexibility in monthly premiums payment plan and use of technology to increase access to payment points. Further study need to evaluate the content of health financing policy in Botswana measured against the World Health Organization Universal Health Coverage conceptual requirements for Low and Middle Income Countries.

**Electronic supplementary material:**

The online version of this article (doi:10.1186/s12913-016-1657-2) contains supplementary material, which is available to authorized users.

## Background

Financing population-based health care is a major global public health challenge because approaches for collecting revenues, risk pooling, and providing or purchasing of health services strive to meet population health needs [1]. In 2010 it was estimated that about 150 million people globally experienced catastrophic health care expenditure annually; whereas in Low-and Middle Income Countries (LMIC) catastrophic out-of-pocket expenditure for health pushes around 100 million people into poverty annually [2]. Health financing experts argue that country specific politics and economic situations determines health financing policy [3–5] which is central for advancing Universal Health Coverage goals [3].

Universal Health Coverage (UHC) also referred to as Universal Health Care Coverage, Universal Health System and Health Coverage [6] is not a new concept. It is embedded in the global call for health as a fundamental human right and well stipulated in the World Health Organization (WHO)’s Constitution [7], in Alma Ata declaration [8] and in Health financing reforms [9]. Universal Health Coverage (UHC) refers to a situation where all people who need health services such as curative interventions which addresses ill health [10]; and population-based health services which tackles Social Determinants of Health and risk factors for non-communicable diseases [11], receive them without experiencing financial hardship [2, 12].

**Fig. 1:**
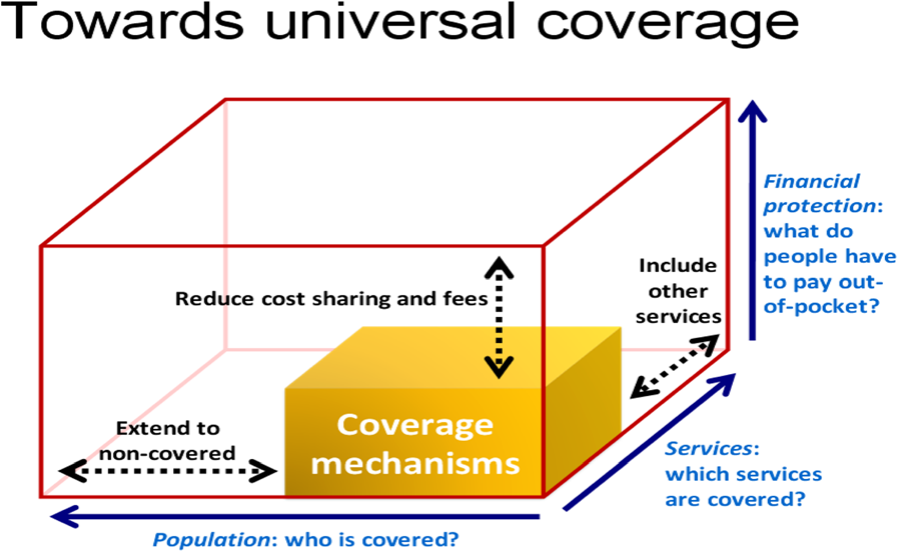
Universal Health Coverage conceptual sketch [13]

UHC concept is within the human right for health framework which advocates for equitable access to health services to everyone [7]. Furthermore, the UHC concept has a tremendous public health, health economics and humanitarian connotations [6] which altogether call for inclusiveness and expansion of health services coverage and financial protection to all residents with special emphasis on protection of the poor and vulnerable population groups. The WHO measures the UHC concept in a three-dimensional cube with three elements: population (who is covered), services (which services are covered), and direct cost (proportion of the cost covered) as depicted in [13] (Fig. 1).

Based on the WHO three-dimensional cube measurements of UHC, it appears that most high income countries such as German, United Kingdom, Canada and Australia have achieved UHC goals through equitable health financing mechanisms [14]. Low and middle income countries (LMIC) started the journey towards Universal Health Coverage goals in 2005 following the 57th World Health Assembly’s resolution number 58:33 which urged member states to reform health financing arrangements towards UHC [1]. Currently with regards to the WHO three-dimensional cube, Ghana through National Health Insurance scheme [15] and Rwanda through Community Based Health Insurance schemes [16] are among few LMIC which have successfully developed a health financing system that advances the goals of Universal Health Coverage concept [17].

Most studies explored tools and methods to measure progress towards Universal Health Coverage [12, 18] thus concluded that lack of consensus on international benchmark for developing and comparing UHC progress is a major drawback for advancing UHC goals [19]. Other studies explored health financing typologies that are potential for advancing UHC goals [5]. These typologies include Tax-based financing where funds are generated from general taxation and health services are freely provided (e.g. UK, Denmark, Sweden); National health insurance model where funds are generated through National health insurance and Private health insurance (e.g. Canada, Australia, Italy, Ireland); Social health insurance plans where funds are generated from employed individuals, commercial corporations and government contribution (e.g. Austria, German, Switzerland); Private health insurance (e.g. USA); and hybrid financing typology which incorporates features of tax-based and insurance models for funds generation (e.g. Japan, Korea, Netherlands, and Belgium) [5, 20].

Among emerging economies like Brazil, the Russian Federation, India, China and South Africa (BRICS) with about 40 % of Global population, studies shows that Brazil adopting tax-based financing, Russia adopting social health insurance, India adopting a hybrid financing typology and South Africa adopting National Health Insurance model [21]. Empirically health financing policy remains central for advancing Universal Coverage goals [3]. For instance introduction of Social Health Insurance plan in German in 1873 [22]; social protection policies in Chile in 1924 [23]; and United Kingdom in 1942 [22, 24] extensively forms a benchmark for 21^st^ Century health financing reforms towards achieving the WHO Universal Coverage three-dimensional goals. Moreover studies exploring the WHO Universal Health Coverage (UHC) concept showed a strong relationship between population health needs and Social Determinants of Health. For example transport cost and healthcare cost at Private facilities deters efforts towards achieving UHC goals in Malawi [25].

Empirical literature indicates that different conceptual terminologies, such as “health coverage schemes” [26], “health insurance schemes” [27], “health coverage enrollment” [28] has been used interchangeably to refer to the same concept .i.e. health insurance schemes. Although some studies have shown that health coverage schemes increases access to health services [29]; improves personal health seeking behavior [30]; and assures availability of quality health services among insured individuals [31]; however other studies have shown indirect relationship between knowledge on health coverage schemes and its enrollment [26, 27, 31–35]. Further studies indicate that health insurance enrollment is determined by socioeconomic status and ability to pay [37, 38]. For example only 21 % of ‘enrollees’—i.e. individuals registered in health coverage schemes—were from low socioeconomic status (SES) in Ghana [37], and only 43 % of ‘enrollees’ were from lowest salary category were insured South Africa respectively [38].

Furthermore, unaffordable monthly premium, regular shortage of drugs, and people’s mistrust to insurance providers were found to limit health coverage schemes enrolment in Uganda [31]. Other studies have established that Willingness To Pay (WTP) is integral for setting insurance premium [39]. Moreover, means testing (MT) and proxy means testing (PMT) measures have been indicated as potential strategies for identifying vulnerable population thus guide setting of insurance premium—i.e. monthly contribution for insurance coverage—and insurance exemption in order to enhance equitable health coverage schemes [40].

## Methods

### Study context, setting, design, study population, sampling and data collection

#### Study context

Botswana is a land-locked country located in the Southern Africa region. It has an estimated population of 2,021,000 and GDP is US$ 14.79 per capita [41]. Life expectancy is estimated at 54.4 years; infant and under-five mortality rates are 54/1000 and 76/1000 live birth respectively; and maternal mortality ratio is 193/100,000 live births [42]. Botswana spends 17.7 % of annual budget for health services [43] which is above Abuja target of 15 % for African countries [44]. Health care expenditure in Botswana are highly subsidized and financed through government taxes (68 %); health insurance schemes (21 %); donors (12 %) and out-of-pocket expenditure (4.2 %) [45]. Nevertheless low out-of-pocket spending for health in Botswana at 4.2 % [45] suggests high risk of Catastrophic Health Expenditure amongst poor population group [46], thus illustrates gaps in the country’s health financing policy towards achieving UHC goals stipulated in the WHO three-dimensional cube.

Alarming prevalence rates of HIV/AIDS at 21.9 % among adults aged 15 to 49 years [47]; high TB prevalence rates estimated at ≥ 300 cases per 100,000 populations [48]; increasing risk and prevalence of non-communicable diseases [49]; and advancing medical technology [50] collectively exerts enormous pressure on fiscal sustainability for UHC agenda in Botswana. Although about 84 % of the entire population in Botswana access health care within 5 km radius [51] thus suggests existence of the infrastructure for advancing UHC conceptual goals, only 17 % of the general population and 42 % of employed individuals have health insurance coverage [52]. Limited health insurance coverage among general population and employed individuals raises concerns on the design of health insurance scheme particularly with regards to efforts towards achieving UHC conceptual framework goals [13].

Most intriguing is the evident inequity of financial resources allocation between curative services and cost-effective public health interventions. For instance, during 2007/08 and 2009/10 fiscal years, hospital curative services were allocated 53 % of Total Health Expenditure (THE) whereas population-based services received only 9 % of THE [45]. Inequity in financial allocation deters efforts towards expanding health services coverage particularly expanding cost-containing strategies population-based health services geared towards disease prevention and health promotion interventions [53]. Inequity in financial allocation between curative and population-based health services further derails initiatives to tackle Social Determinants of Health [10, 11].

Globally to the best of researcher’s knowledge, there is no published study that explored population perspectives regarding financing population-based interventions through health coverage schemes in order to advance UHC goals. Despite most studies utilizing WHO Universal Health Coverage conceptual framework [13] to identify measurable indicators for UHC concepts [12], to the best of researcher’s knowledge there is no published study of similar nature conducted in Botswana. Most published studies on health coverage schemes investigated understanding and perspectives on financing curative intervention through health coverage schemes [30–36]. Nevertheless there is no study of similar nature which has been conducted in Botswana despite available indicators showing low enrollment in health coverage schemes [52].

Further to that, among Sub-Saharan Africa countries Botswana presents an exceptional commitment and surpassed Abuja declaration target of 15 % on healthcare spending for Africans countries by spending 17.7 % of Total Health Expenditure [43, 44]. However, only Ghana and Rwanda have developed financing system that advances the three-dimensional UHC conceptual goals by allocating 12.5 [15] and 16 % [16] respectively. Therefore literature is limited in explaining why and how Botswana does not have a health financing system that advances UHC goals as defined by the WHO three-dimensional conceptual framework. Therefore this study is set to fill the knowledge gap on health financing literature in Botswana by exploring perspectives of employed individuals on financing population-based interventions—i.e. health promotion and diseases prevention interventions—as the country gears-up towards achieving Universal Health Coverage goals.

## Study setting

We conducted the study in Ghanzi district, a semi urban multi-ethnic town situated at Northwest region, Botswana. It has widely dispersed population projected at 36,211 equivalents to 1.8 % of the country’s population [54]. The district has a total of 16 health facilities comprising a network of 10 health posts, 3 clinics and 1 primary hospital run by the government where curative services are provided based on need; and two (2) private-run health facilities where curative care cost are covered through out-of-pocket payment or insurance coverage. Population-based interventions are largely financed through government coffers and donors; and periodically provided through health education and promotion campaigns conducted by government facilities [51]. Creswell indicates that, exploration of opinions and meaning from individuals with diverse social demographic background and experiences on a phenomenon generates multiple realities of the phenomenon [55]. Thus the study site was selected due to its multi-ethnicity nature and diverse demographic background which assures multiple social reality regarding financing population-based health services towards achieving Universal Health Coverage.

### Study population and sampling

Within the qualitative framework of this study, we purposefully gathered information-rich data regarding perspectives of employed individuals on financing health promotion and diseases prevention interventions towards achieving WHO Universal Health Coverage goals. Purposeful sampling technique [55] and snow-balling sampling technique [56] were utilized during sampling and recruitment procedures. The study sample included English speakers individuals aged 18 years old and above residing in Ghanzi district for at least 5 years, employment status, insurance status and awareness regarding health coverage schemes. The use of English language was preferred because it is the official language in employment setting in Botswana (57). A minimum of 5 years residence in Ghanzi district was important because it assured that study participants have adequate knowledge regarding challenges facing the community upon seeking health services. Conversely, employed individuals over age of 18 years who were unknowledgeable regarding health coverage schemes, unemployed individuals who are either insured or uninsured, English speakers who reside in Ghanzi for less than 5 years, as well as non-English speakers residents were excluded from the sample. Moreover, since employment does not guarantee health care coverage, half of the eligible sample (*N* = 8) had insurance and half (*N* = 8) had not. Further to that, the sample was split into half by gender. A total of 15 eligible participants completed the interview. Nine (9) were public employees and six (6) were private employees. Among them, 7 were females and 8 males. Half of the respondents (*n* = 8) had insurance coverage, others (*n* = 7) had no insurance Table 1.

**Table 1:** Respondent’s profile compared with researcher’s positionality

Respondent characteristics	Researcher’s positionality	Respondent’s profile
Age		20–29 (*n* = 3)
	X	30–39 (*n* = 6)
		40–49 (*n* = 5)
		50–59 (*n* = 1)
Gender	X	Males (*n* = 8)
		Females (*n* = 7)
Level of education		Secondary school certificate (*n* = 2)
		College diploma (*n* = 7)
	X	Undergraduate degree (*n* = 6)
		Post graduate degree (*n* = 0)
Employment status	X	Public employees (*n* = 8)
		Private employees (*n* = 7)
Insurance coverage status		Insured (*n* = 8)
	X	Uninsured (*n* = 7)
Duration stay in Ghanzi district	X	≥5 years (*n* = 11)
		≥10 years (*n* = 4)
Duration enrolled in health insurance	X	None (*n* = 7)
		≤5 years (*n* = 3)
		≥6 years (*n* = 5)

### Data collection

Data was collected from November 2014 to January 2015 within the context of exploratory qualitative study with the purpose of informing formulation of strategies for financing population-based intervention towards Universal Health Coverage (UHC) agenda. Employed individuals were selected because they are potentially the primary source for financing health care through government taxes and insurance premium contributions. Thus their experiences with health coverage schemes may inform formulation of sustainable health financing policies, programs and strategies that assure fairness of financial contribution for all towards advancing UHC goals in Ghanzi district and the country at large.

The principal researcher conducted all interviews with the target population in English [57]. The interview guide comprises of the following topics: i) Demographics including current and previous health coverage status and employment; ii) Understanding and perspectives on health coverage schemes in Botswana; iii) Understanding and perspectives on Universal Health Coverage; iv) Recommendation for change. The interview topics were informed by literature review on WHO Universal Health Coverage three-dimensional conceptual framework [13], inequity allocation of financial resources between curative and cost-effective health promotion and diseases prevention interventions [45], and levels of insurance enrollment in Botswana [52].

Two pilot interviews, one with insured and one with uninsured employed individuals were conducted to enable familiarization of the data collection tool and shaping researcher’s expectation of the field work [58]. The interview duration varied between 30 and 45 min. The in-depth interviews started with reading the introduction part of interview guide, thereafter the first question asked was: “have you ever heard of health coverage schemes?” Thus enable further screening of eligible participants and easing into the conversation. Most of interviews (*n* = 10) took place at respondent’s home, while some (*n* = 5) were conducted at participant’s office according to participant’s preferences [59]. All in-depth interviews were recorded using a digital recorder inbuilt in iPad2 device.

### Ethical consideration

Ethical clearance of the study was obtained from Botswana Health Research Secretariat (BHRS) and University of Liverpool, UK due to researcher’s affiliation to the University. Written informed consent was obtained from all participants prior to the beginning of the in-depth interview. Emphasis was made on voluntary participation and the right to withdraw from the study at any point without consequence [60]. All eligible participants consented to and participated in the study. During interviews transcription, the researcher’s removed all potential identifiers that could facilitate participants tracing.

### Data analysis

Thematic Content Analysis (TCA) technique [61, 62] was chosen for analyzing data. The technique is important because it enhances flexible categorization of interview data into commonalities and differences in order to generate themes (ibid). Furthermore, TCA allows unique presentation of respondent’s perspectives through context specific theoretical assumption.

Data driven analysis guided development of coding scheme [63]. However the research question and objectives had pre-set elements that initially guided coding the scheme. Therefore, codes were not predetermined, but emerged from respondent’s opinions on study phenomenon.

Analysis was conducted manually using Microsoft Word 2007 document. Systematic identification, coding and interpretation of participant’s accounts were made. The initial step was formatting transcribed interview data into tables. The next step was to develop a code-subtheme-thematic code book which included preset and emergent themes with definition of each individual theme and subthemes per theme. The researcher established numerical codes to each subtheme and themes which were added in a separate column of each table. Thirdly, data categorization and migration were conducted to establish pattern. Categorization was critical for code validation in order to establish potentially extreme cases and confirm emergent themes [64] (Additional file 1).

### Methodological consideration

Every participant’s response potentially provided a range of themes and subthemes, thus a smaller sample size was sufficient to achieve data saturation [65]. The first author’s background as employed, educated, uninsured individual and a resident at Ghanzi district for more than 5 years was similar to most of participants’ profiles. He consistently reflected on his own values in order to avoid clouding data collection and analysis [66]. The second author did not have any direct contact with the participants and was involved in aspects of planning, data analysis and authoring the research. Participants were aware of first author’s position and freely expressed their perspectives on financing population-based health care towards UHC.

The first author assumed a continuum insider and outsider roles in order to maximize the advantages while minimizing potential limitations of each role [67]. Through the insider role, the researcher was able to understand participant’s culture and practices; facilitate natural interaction with participants; and favorably enhanced confidential relationship [68]. As an outsider, the researcher acknowledges limited understanding about subcultures of the study population because he is not a native from the study setting [69, 70]. Thus, throughout data collection and analysis, the first author aimed to understand subcultures and how they influence perspectives on financing population-based interventions through health coverage schemes towards achieving WHO UHC conceptual goals. Reflexive, non-judgmental and empathetic positionality [68–70] coupled with open acceptance by gatekeeper’s enhanced cross-cultural divides. Therefore, valid results are produced by both insiders and outsiders positions, thus reflecting on each researcher’s position provides strength of the study [59, 69, 70].

Strategies applied to maintain scientific rigor [71] included member’s checking and constant comparison of participant’s accounts and literature throughout data collection and analysis processes; thick description of study phenomenon, setting and sample [59, 71]. In addition, participant’s audit check and secondary researcher’s critical analysis of data was instrumental for scientific rigorous [69, 71]. Essentially, important steps conducted for this study have been detailed described in this paper including study setting, participant’s recruitment procedures as well as data collection and analysis. This enhances transparency and transferability of the study within different settings. Selected quotations from the transcripts have been incorporated in the results section to demonstrate study key findings and to give a voice to participants.

## Results

Table below presents the profile of the sample population Table 2.

**Table 2:** Distribution of Sample population by Age group and Sex

	Age-group (years)				TOTAL
	20–29	30–39	40–49	50–59	
Number of males	0	4	3	1	8
Number of females	3	2	2	0	7
TOTAL	3	6	5	1	15

A total of 15 employed individuals including 8 males and 7 females participated in the study. Their age ranged from 23 to 59 years with a mean age of 31 years, and median of 36 years. Nine (9) were public employees and six (6) were private employees

A total of 15 employed individuals including 8 males and 7 females participated in the study. Their age ranged from 23 to 59 years with a mean age of 31 years, and median of 36 years.

Five main themes emerged from analysis of qualitative data:

- Insurance coverage issues.
- Universal health coverage issues.
- Contributing for population-based health care issues.
- Efficiency governance of resources for health issues.
- Sustainability of contracted employees on health coverage schemes.

Relevant interview accounts are quoted whenever necessary. Quotations adopts a coding system established by a researcher such that “R” stands for “Respondent” and “I” stands for “Interviewer”. All “R” are followed by numerical numbers, for instance “R01”, “R02”… “R16” indicates “Respondent #1”, “Respondent #2”…to “Respondent #15”.

### Insurance coverage issues

Irrespective of gender, age, employment and insurance enrolment status, participants showed a broad understanding of health insurance concept, functions and operations.

Regarding awareness on public and private health insurance schemes, participants indicated that, public and private health insurance schemes are optional health coverage arrangements available to all government and private employees.

*R05: “…much I know about it, is an optional facility offered to employed individuals and those in contract to take part.”*

*R08: “If you want to be covered, you have to join health insurance scheme which can cover anything that you encounter maybe road accident, or maybe any help on health problem. You can also include some of your family members and they will be covered.”*

Despite demographic indicators, participants felt that coverage schemes are sector specific.

*R04: “Private [Insurance schemes] is actually for private employee like me, you know every health scheme or company have their different packages and what not, so everybody tries to offer the best for health.”*

*R11: “What I have heard about public health insurance schemes, Is that, this is an arrangement where the employer pays some percentage and then the beneficiary himself, pays a certain amount to access health services. Private health insurance schemes deals with the people who are employed in the private or even those are self sponsored.”*

With regard to roles of health coverage schemes: despite participant’s demographic background, employment and insurance status, participants collectively indicated that the fundamental role of health coverage schemes is to provide financial protection against catastrophic spending when one falls ill.

*R03: “…basically to help the insured cover the cost for medical treatment when they fall sick. When you fall sick many times the cost of the treatment is much higher than the premium that you pay. So, instead of paying this cost…then you can access those services at a lesser fee.”*

*R06: “Sometimes there are some diseases that you cannot be helped at the public hospital. Then you have to go to private hospitals where the insurance will pay for you.”*

Comparatively, all participants despite insurance and employment status are knowledgeable regarding roles of health coverage schemes.

Participants showed mixed perspectives on insurance operations particularly monthly premiums contributions. All public employees’ participants indicated that only in the public scheme where health coverage schemes are subsidized both employer and employee contribute 50 % of monthly premiums. A few private employees’ participants reported similar perspectives. Contrarily, most of private employees’ participants indicated that their monthly subscriptions are unsubsidized.

*R09: “…the private ones I think you pay them through private insurance companies… they do not involve the government”; in the government “I pay through my salary from the government to them…and then the government somehow has a share.”*

Regarding health services coverage through health insurance schemes, irrespective of demographic indicators, participants indicated that health insurance schemes mostly covers of ill-health conditions.

*R01: “The services that are accessed, most of them are curative that is when you are sick, that is when you will be covered.”*

However, varying perspectives emerged across demographic indicators regarding coverage of population-based services such as prevention and promotion services. Few participants were unsure whether these interventions are covered through health coverage schemes; others indicated that insurance schemes do cover these services; some indicated that they are not covered through coverage schemes.

*R10: “I’m not sure whether they are covered because I’ve never received such messages from them.”*

Responding to a question on advantages of health coverage schemes, all participants despite insurance coverage and employment statuses collectively indicated that insurance coverage increases access to health care services, assures timely and quality of care provided.

*R03: “…you don’t have to worry about the cost of treatment whenever you fall sick. Because you know, money sometimes is very difficult to keep and have them. Anytime we fall sick, that burden [financial] is covered by medical schemes.”*

However, participants reported mixed concerns regarding limitations of health coverage schemes. Some participants felt that, health coverage schemes are discriminatory because low-scale employees and unemployed individuals are systematically excluded.

Low and Middle premium benefit enrollees categorically experiences limited services coverage, thus financial and services coverage are not guaranteed.

*R02: “Is like you are paying for 6 years there and maybe now you are sick, you want a big operation and they say the money is less.”*

Others argue that, when an enrollee has contributed for 2–3 years without falling ill and thus not utilizing health scheme, there is no refund.

*R06: “We are keeping on contributing if we are getting services…For instance, if you are not sick…what are they doing with the money?.”*

The concern regarding unequal healthcare opportunities among population groups emerged throughout participant accounts. Thus social determinants of health such as accessibility to health services, level of education and income, employment status, working environment, housing, place of residence, food availability, and social support emerged as key determinants for population health status. Comparatively, participants felt that health coverage schemes are not designed to tackle social determinants of health such as diet, socioeconomic status, housing and employment.

*R04: “I believe these health insurance schemes they just wait for us to fall sick.”*

Furthermore, participants felt that financing population-based health care is essential for surveillance, early diagnosis and management of chronic non-communicable conditions such as mental health, disabilities, hypertension and diabetes.

*R01: “Some children …they are born with disabilities. Only to find that these disabilities if they were taken care of, or controlled at an early stage, they could be sorted out.”*

### Universal health coverage (UHC) issues

Irrespective of participant’s age, gender, level of education, employment and insurance statuses, they collectively showed limited understanding of UHC.

Regarding contributing towards UHC, participants collectively felt that contributing for population-based interventions is a good idea because prevention is better than cure. However, some showed concerns on its implementation.

*R05: “I think is a good idea to be all inclusive. Is good idea but am not sure of what logistics could be applied there.”*

*R11: “I think that could be a noble idea. Sometimes when we are already trapped with some ill health, it is a bit difficult to get cured, and therefore becomes very much expensive for both government and the individual himself because the quality of life is already compromised.”*

Some participant indicated that UHC concept is grounded in social solidarity and cross-subsidization concepts, thus felt that majority of employed individuals would be willing to embrace the idea.

*R07: “I think it is a great idea, so I am positive that a lot of people…maybe not everybody, but I think a lot of people…more than 50 % of people would love to make a contribution…”*

Participants across demographic indicators collectively indicated willingness to contribute for UHC. Regarding amount to contribute, some opted to contribute certain percentage of salary; others opted to contribute a particular amount of money. Those willing to contribute a particular amount are willing to contribute between 10 and 300 Pula per month. Nevertheless, participants were not specific on percentage contributions.

*R04: “…in Setswana we have this thing saying - it doesn’t matter how much you pop-out as long as it is something - it gonna make a difference.”*

Across participant’s demographic profile, factors such as limited understanding of UHC concept; negative attitude towards UHC; shortage of human resource; individual’s budget constraints; potential abuse of health services delivery at point of care; and political/health policy environment was identified as potential barriers for UHC.

*R11: “The barriers obviously, the one comes into my mind is politics. Because obviously politics, some might have say I want this policy in good faith, others will take it for their own advantage, some will just decide to block it because it is a good idea, so they will just want to delay that so that they can have a political mileage out of it”*.

Regarding tackling barriers for UHC, majority of participants opined that public education is critical for addressing knowledge gap on UHC. Public consultations, research and benchmarking were identified for tackling political and health policy barriers. Whereas addressing individual’s negative attitude and potential abuse of health services delivery requires intensive public education and consultations.

*R01: “revisiting the current healthcare policy, the act…and there are some pathways that one need to employ here, like here we have ward meetings, ward leaders, elders and stuff, not even forgetting the youth.”*

*R07: “Consultation is best. Consultations includes workshops, seminars and all that, and educating the employers and all that.”*

Furthermore, all participants felt that strategic partnership/collaboration between public and private funders are instrumental for funds mobilization, efficient allocation and paying for population-based health services.

*R14: “there is a need of partnership between the government and the private sector. The government cannot afford alone really, but I think from the private sector they are not doing enough to support the government, there seem to be profit oriented.”*

Nevertheless, participant’s had no opinion on how to address individual budgetary constrains as a UHC barrier.

### Financing population-based health care issues

Some participants felt that the existing health financing arrangement should be strengthened because it allows the government to tackle social exclusion and health disparities among population groups.

*R05: “I would suggest probably government should increase the financial budget in the health sector as the basic human need.”*

Other participants opined that the government as a custodian of national health insurance funds, may consider investing those funds in off-shore investments and then, returns on investment can further finance the national insurance fund.

Another participant viewed that private sectors are potential source of revenue for population-based interventions.

*R04: “I think best way is to firstly approach the big organizations, the private companies because they are the ones who wants to be there in the forefront. And then yes, um also approach individuals.”*

While another suggested that increasing financial resources for health requires compulsory health coverage scheme arrangement.

*R15: “…the multination companies which are hiring most of the population in Botswana should provide these schemes to their own worker because, currently the private sector they are not…is not compulsory for them to provide coverage for their workers.”*

### Efficiency governance of financial resources for health

Across demographic indicators, all participants felt that health coverage schemes need to consider expanding population-coverage, services coverage and provision of financial protection for entire population.

All participants felt that population groups with high socioeconomic status (SES) experiences better health coverage than those on low and middle SES. Thus respondent opines that, health coverage arrangement need to expand population coverage to entire population.

R15: *“…no one will want their families to be excluded because our health is not only us, your health is including even the family members, and so if they are covered and you are covered, it is going to become cheaper to us [the employed]*

Majority of participants felt that extensive provision of curative services among public and private providers limits efficiency governance of resources for health in improving population health.

*R10: “There are chronic illnesses if left unattended people will be losing life earlier than expected. And some of them also spread. So, it is important all the diseases are covered to control the spread and save life.*”

All participants indicated that the existing fragmented health financing mechanisms creates social and health disparity among population groups. Thus, opined that coverage schemes needs an inclusive approach to cover entire population.

*R06: “…if there is a new arrangement where we can be able to pay then it is okay…or if we can’t then the government should pay for us if we are not working.”*

### Sustainability of insurance coverage issues

Few participants showed concerns regarding sustainability of health coverage schemes among contracted employees in public and private sectors. When contract expires, the employer withdraws employee’s monthly contributions resulting in termination of insurance coverage.

*R06: “When we were there, the contract finished. So, we had to leave the job and then we were home”; “In the private we work through the contract, if there is no contract, there is nothing. If [a contract is there] that is when you will be insured. But this time I said no no no, I am popping out a lot of money but at the end that money is gone”*.

Comparatively, in the context of sustainability of monthly premium contributions, only contracted employees in public and private sector considers health coverage arrangement unrealistic Table 3.

**Table 3:** Summary of key findings

Health coverage schemes
1. All participants despite demographic background including insurance coverage status showed a broad understanding on the concept of health insurance, its functions and operations. 2. Majority of participants stated concerns regarding exclusion of population-based health services from health coverage schemes arrangement. 3. All participants expressed concerns regarding health coverage schemes that cover only payroll employees. The arrangement systematically excludes low-income earners and unemployed individuals thus creating social and health disparities. 4. All participant felt that population health is determined by the social and structural environment where a person resides.
Universal Health Coverage
1. All participants’ showed limited understanding of the concept. However, upon a brief description, respondents showed willingness to embrace it. 2. All participants felt that UHC agenda is an inclusive approach for addressing population health needs.
Financing population-based health services.
1. All participants indicated that, the government should play a central role in financing population-based health services. 2. Some participants indicated that, a compulsory health financing arrangement is vital for enhancing public-private partnership in financing population-based services through UHC.
Efficiency governance of financial resources for health
1. All participants felt that, improving efficiency in financial resources governance requires strategic integration of financial resources and service delivery.
Sustainability of insurance coverage among contracted employees.
2. Majority of the participants felt that, retention of contracted employees in the existing health coverage schemes requires review of the national health financing policy.

## Discussion

### Health coverage issues

The study aimed at exploring employed individual’s perspectives on health coverage schemes. Results show that majority of participants are knowledgeable about health coverage scheme concepts. Thus, suggesting the concept is well understood among insured and uninsured employed individuals. Identical results were outlined in a qualitative inquiry conducted in Maliando district, Guinea-Conakry [26]. Unlike the study in Maliando district where the sample size was 137 villagers, this study recruited a total of 15 participants. The sample size was adequate to generate multiple realities regarding knowledge on health coverage schemes [56, 65].

Participant’s knowledge about health coverage schemes appears insignificant in increasing health coverage enrolment among employed individuals. Despite awareness, only 17 % of employed individuals and 42 % of the entire population has medical aid cover in Botswana [52]. Therefore, catastrophic health expenditure is likely to keep-on rising [46] and the country will not realize the dream for fiscal sustainability in health care. Findings suggest that optional health coverage policy is unsuitable for advancing inclusive Universal Health Coverage agenda thus it is prudent to consider mandatory health coverage policy.

Majority of participants are knowledgeable on roles of health coverage schemes. Despite differences in study setting and design, the findings are similar with results from a cross-sectional descriptive study conducted in Rubabo County, Uganda [31]. However in an era of epidemiological transition characterized by increasing risk factors and prevalence of non-communicable diseases and conditions [49, 51] as well as increasing financial austerity [42, 43, 50] whereby insurance schemes largely covers ill-health conditions through contracted private health facilities, participants remains puzzled why health insurance schemes do not introduce health coverage arrangement for population-based health services through private health care institutions.

Limited understanding regarding operations of health coverage schemes was evident among majority participants. Similar findings were reported from a quantitative survey conducted among National Health Insurance Scheme users (NHIS) in Minna-Niger state, Nigeria [32], in a mixed study conducted in Nairobi, Kenya [33], and in a case study evaluation carried out at two selected CHI schemes in the Ishaka and the Save for Health Uganda (SHU) schemes in Uganda [34]. Limited understanding on operations of health coverage schemes appears to be context specific. For example , Nigeria and Kenya has mandatory health coverage arrangement while Botswana has optional health insurance schemes. Nevertheless, despite contextual differences, the findings suggest increasing need to scale-up information dissemination regarding insurance operations in order to increase its uptake.

Lower salary scale employees do not opt to enroll in health coverage schemes. Identical findings were evidenced from a cross-sectional study conducted at Ashanti region, Ghana [37], and from a multi-stage quantitative study conducted in South Africa [38]. The findings suggest unequal opportunity to health insurance benefit among population groups. In this study however, employees at lower salary scale, who opted to enroll in coverage schemes, could only afford to contribute for low and middle premium benefit packages where financial protection and health services utilization are highly limited. Empirically, the design of health coverage schemes in Ghana, South Africa and Botswana greatly excludes health needs of underprivileged population groups’ thus unfairly yet unintentionally exacerbates social and health inequalities.

### Universal health coverage issues

Findings point out that majority of participants *(n = 14)* have limited knowledge regarding the WHO Universal Health Coverage concept. Thus UHC appeared as a new concept among employed individuals. Furthermore, despite the call to reform health financing policy towards UHC [1], Botswana government appears not to have taken significant measures towards inclusiveness arrangement for health insurance schemes.

Moreover the Abuja declaration required African countries to spend 15 % of annual budget for health [43]. Only Ghana which spends 12.5 % [15] and Rwanda which spends 16 % of annual budget [16] has health financing system that advances UHC. However, despite spending 17.7 % of annual budget for health [42], Botswana does not have a health financing system to advance UHC. Thus suggesting that Botswana requires political commitment to drive the UHC agenda.

Further results show that insurance schemes in Botswana cover most of ill-health conditions thus increase utilization of curative interventions. Identical results were reported by Lagarde & Palmer [35]. Intentions of health coverage schemes appear to results in unfair, systematic and unacceptable exclusion of financing diseases prevention and health promotion interventions. Thus creating inequity fragmentation between health services financing and services delivery upon responding to population health needs.

Majority participants *(n = 14)* showed willingness to contribute to UHC through health coverage schemes. A mixed study conducted in Liwale district, Tanzania reported similar findings [36]. Hence implies that Botswana has a social-cultural background that embraces social and economic risk protection values. These values are integral for designing and implementing a financing system that advances UHC agenda. Therefore, despite differences in study setting, design and sample characteristics between these studies, findings and literature has deepen our understanding regarding potential gaps in the design features of health coverage schemes in Botswana which hence impedes increasing enrollment of potential clients.

### Financing population-based health care

The findings points out that funding for population-based interventions should be derived from various sources. However the government should remain a major source because it effectively tackles social exclusion and health disparities. Thus it appears that, health care arrangement in Botswana assumes social protection policy to ensure all citizens receives health care services based on need; not on ability to pay. Similar results are evident in United Kingdom [22, 24] and in Chile [23]. Nevertheless, the context of delivery of health care based on need markedly differs between these countries because in the United Kingdom for instance, health services are financed through general taxation and freely provided at point of care [22, 24]. In Botswana health services are highly subsidized and largely financed through government taxes (68 %), insurance schemes (21 %) and out-of-pocket (4.2 %) [45].

Importantly, health financing arrangements in United Kingdom and Chile has developed a system that advances UHC goals. In contrary Botswana health financing system hasn’t adequately evolved to advance UHC goals. Hence there is a need in Botswana to reorient health coverage schemes from optional variant to mandatory variant of health coverage policy such that all employees and employers at public, private and informal sectors participate in contributing financial resources for health as the country gears towards achieving Universal Health Coverage.

### Efficiency governance of financial resources

The findings indicate existing inefficiency and inequity in allocation of financial resources between curative and population-based health services (i.e. diseases prevention and health promotion services). Identical findings were reported from a study on allocation of financial resources between hospital services and public health interventions conducted in OECD countries [72]. Inefficiency and inequity in resources allocation between health services indicates marginalization of cost-effective public health interventions. Moreover, the findings impliesthat policy makers and planners are unfairly skewed towards financing highly expensive curative interventions instead of allocating more resources for cost effective population-based interventions.

### Sustainability of insurance coverage among contracted employees

Retaining contracted employees in health coverage schemes upon contract termination is a key finding. Therefore suggests existing health coverage arrangement for contracted employees in Botswana exhibits similar patterns as health coverage arrangement for informal sector employees observed in Kenya and Tanzania [33, 36]. The Kenyan and Tanzanian health coverage schemes has structural design and implementation features that appear to unfairly exclude individuals with irregular income, hence limits coverage expansion for needy population and thus exacerbates financial risk for health. Therefore unethically contributes in pushing vulnerable and unpriviledged population groups into catastrophic healthcare expenditure and poverty [2].

Thus, while the large proportion of the population in Botswana is outside the formal employment sector [52], it is critical that reforming health financing policy including the structural design and implementation features of health insurance schemes remains integral for expanding financial protection to everyone particularly vulnerable population groups.

## Limitations

This study has generated significant value into perspectives on financing population-based health care through UHC among employed individuals in Ghanzi district, Botswana. However since majority of the population in Botswana are not in the formal employment sector [52] inclusion of informal sector employees could generate even more diverse and rich data thus deepening our understanding on arranging for health coverage schemes in Botswana. Additionally, a qualitative inquiry which combines semi-structured interviews and Focus Group Discussion would provide more rich and diverse data as well as allowing data triangulation and deviant cases analysis thus increasing credibility of the findings.

## Implications for public health practice

Health coverage schemes in Botswana systematically excludes majority of the population and highly excludes population-based healthcare interventions. It therefore indicates that majority of the population experiences a hidden catastrophic health care spending on one hand [45, 46] and increasing risks and prevalence of non-communicable diseases on the other [49, 51]. Although most of participants in this study indicated a willingness to contribute for population-based health care through health insurance schemes towards achieving Universal Health Coverage goals, participants indicated limited understanding regarding the design of health insurance schemes, and how that design would expand health services coverage and population coverage as well as assuring financial protection against catastrophic healthcare expenditure. This finding is of high relevant to public health practice because it indicates need to increase financial resources for health services, expand population coverage particularly the poor and disadvantaged population groups and subgroups, and expanding financial protection for all in line with the WHO UHC conceptual framework [13].

Informed by Social Ecology Model (SEM) [73, 74] that human health and illness is influenced by the interplay of diverse individual and environmental factors, therefore integration of health coverage schemes based on SEM’s five interconnected levels **i.e.**individual, interpersonal, institutions and organizations, community, structures and systems - is of uttermost important. The new arrangement will assure incorporation of genetic factors and individual behaviors as key determinants for increasing health coverage schemes enrolment, financial protection and services coverage.

Through SEM health coverage schemes design features will enable strategic financing of health promotion and diseases prevention interventions through programs carried at interpersonal level to address cultural practices that jeopardizes public health [72]; and financing programs carried at health promotion settings for advancing UHC agenda. The study furthers that primary requirement for overcoming inefficiencies in health financing policy is political commitment. In most OECD countries, UHC agenda has been achieved through political commitment in reforming health financing policies [22–24]. Political commitment for advancing UHC goals is also evident among Low and Middle Income Countries particularly in Ghana [15] and Rwanda [16] where they have developed UHC financing system. Therefore, while acknowledging Botswana political stability and government commitment on health care spending [43–45, 52], lack of political commitment appears a major drawback for inclusiveness health financing policy towards UHC.

## Conclusion

Globally, most of public health studies on Universal Health Coverage (UHC) and health insurance schemes are focusing on financing curative interventions; the researcher did not find a published study of similar nature conducted in Botswana. This study made a unique contribution by examining perspectives of employed individuals on whether health insurance schemes can be used to finance population-based interventions as a financing mechanism towards achieving the WHO Universal Health Coverage conceptual goals. Participant’s perspectives in Ghanzi district, Botswana affirmed that reformed health coverage schemes are potentially positioned to finance population-based interventions towards achieving UHC conceptual goals. The perspective provides positive indicators for policy makers to integrate population health needs in the WHO UHC agenda. However, a strong understanding of health insurance schemes does not facilitate enrolment in health coverage schemes. Thus increasing enrollment requires strengthening information dissemination using different channels and methods targeting different population groups. Re-designing health insurance schemes to accommodate flexible arrangements for monthly premiums payment including access to payment points and expanding its services coverage to include population-based services is central for advancing UHC goals.

Furthermore, efforts to improve allocation of financial resources between health services requires a priority setting policy guideline that emphasizes equitable distribution of financial resources to address population health needs and Social Determinants of Health. A further study is therefore needed to evaluate the content of health financing policy in Botswana measured against the World Health Organization Universal Health Coverage conceptual requirements for Low and Middle Income Countries (LMIC). The systematic evaluation will inform and guide reorientation of approaches to finance cost effective population-based interventions—i.e. health promotion and diseases prevention interventions—in order to fill context-specific gaps.

## Additional files

Additional file 1: Thematic-index-code book. (DOC 43 kb)

## Abbreviations

LMIC: Low and middle income countries
R: Respondent
SEM: Social ecological model
UHC: Universal Health Coverage
WHO: World Health Organization
