# Supplementary material for: “Perspectives on financing population-based health care towards Universal Health Coverage among employed individuals in Ghanzi district, Botswana: A qualitative study”
Source: BMC Health Serv Res. 2016 Aug 19;16:413. doi: 10.1186/s12913-016-1657-2 (PMC4992196; doi:10.1186/s12913-016-1657-2)
Supplement: Additional file 1: — Thematic-index-code book. (DOC 43 kb) [file 12913_2016_1657_MOESM1_ESM.doc]

Additional information: Thematic-index-code book

| Table 3.2 : Thematic index code book | | | |
| --- | --- | --- | --- |
| **Main theme** | **Subtheme** | | **Codes** |
| Insurance coverage schemes  *Question 2.1 – 2.5* | *Awareness of public and private health coverage schemes* | | - 1. Awareness   2. Role   3. Operations |
|  | *Health services covered by insurance schemes* | | - 1. Health services covered |
|  | *Benefits of health coverage schemes* | | - 1. Accessibility, quality   2. Quality of care |
|  | *Limitations of health coverage schemes* | | - 1. Services provision depends on monthly premiums.   2. Diseases focused   3. Limited services coverage   4. Discriminatory   5. Population health needs unattended |
|  | *Unequal health care among population groups* | | - 1. Socioeconomic status determines quality of life |
|  | | | |
| Universal Health Coverage  *Question 3.1 – 3.8* | *Understanding UHC* | | - 1. Awareness   2. Role of UHC |
|  | *Contributions and response towards UHC.* | | - 1. Willingness to contribute   2. Willingness to cross-subsidy |
|  | *Barriers for UHC* | | - 1. Limited understanding of UHC concept   2. Limited funds   3. Negative attitude   4. Political/Policy environment |
|  | *Tackling UHC barriers* | | - 1. Population empowerment   2. Stakeholder’s consultations   3. Benchmark   4. Research |
|  | | | |
| Financing population-based health care  *Question 4.1 – 4.5* | *Sources of revenue* | | - 1. Sources of revenue |
|  | | | |
| Efficiency governance of resources for health  *Question 4.6 – 4.7* | | *Expanding population coverage* | - 1. Fairness in financial protection for all |
|  |  | *Expanding services coverage* | - 1. Coverage of population-based health care |
|  |  | *Expanding financial coverage* | - 1. Fairness in financial contribution |
|  | | | |
| Sustainability of health coverage schemes among Contracted employees  *Question 2.1 – 2.5* | | *Unrealistic insurance arrangement* | 5.1 Reform health coverage arrangement |
|  | | | |
